# Natural Treatment Systems as Sustainable Ecotechnologies for the Developing Countries

**DOI:** 10.1155/2013/796373

**Published:** 2013-06-26

**Authors:** Qaisar Mahmood, Arshid Pervez, Bibi Saima Zeb, Habiba Zaffar, Hajra Yaqoob, Muhammad Waseem, Zahidullah, Sumera Afsheen

**Affiliations:** ^1^Department of Plant and Soil Sciences, University of Kentucky, Lexington, KY 40546-0091, USA; ^2^Department of Environmental Sciences, COMSATS Institute of Information Technology, Abbottabad 22060, Pakistan; ^3^Department of Biology, Allama Iqbal Open University, H-8, Islamabad 44000, Pakistan; ^4^Department of Zoology, University of Gujrat, Gujrat 50700, Pakistan

## Abstract

The purpose of natural treatment systems is the re-establishment of disturbed ecosystems and their sustainability for benefits to human and nature. The working of natural treatment systems on ecological principles and their sustainability in terms of low cost, low energy consumption, and low mechanical technology is highly desirable. The current review presents pros and cons of the natural treatment systems, their performance, and recent developments to use them in the treatment of various types of wastewaters. Fast population growth and economic pressure in some developing countries compel the implementation of principles of natural treatment to protect natural environment. The employment of these principles for waste treatment not only helps in environmental cleanup but also conserves biological communities. The systems particularly suit developing countries of the world. We reviewed information on constructed wetlands, vermicomposting, role of mangroves, land treatment systems, soil-aquifer treatment, and finally aquatic systems for waste treatment. Economic cost and energy requirements to operate various kinds of natural treatment systems were also reviewed.

## 1. Introduction

 Rightly defined by Mitsch and Jørgensen [1], “the ecological engineering is the design of sustainable ecosystems that integrate human society with its natural environment for the benefit of both.” It involves the restoration of ecosystems that have been substantially disturbed by human activities such as environmental pollution or land disturbance and the development of new sustainable ecosystems that have both human and ecological values. The development of ecological engineering was spawned by several factors, including loss of confidence in the view that all pollution problems can be merely solved through technological means and the realization that with technological means, pollutants are just being moved from one form to another. Conventional approaches require massive amounts of resources to solve these problems, and that in turn perpetuates carbon and nitrogen cycle problems, for example [1].

Currently, economic growth in developed nations, human population explosion in certain areas of Asia and Africa, deforestation, and destruction of natural habitats for the conservation of biodiversity are the biggest challenges for implementing the principles of ecological engineering in most of the developing nations. Currently, economic crunch in many developed as well as developing nations is forcing to implement low-cost natural treatment systems for the domestic and industrial wastewater treatment. In case the technological treatment facilities are installed in many developing countries, the energy input is difficult to be supplied in view of the global energy crisis, and very high operational cost is a bottleneck to their affordability. These all factors are compelling the employment of low cost natural treatment systems for not only waste treatment but also for conserving biological communities in poor nations of the world. The conventional systems that may be appropriate in industrialized regions and densely populated areas with guaranteed power supplies, easily replaceable parts, and a skilled labor force to ensure operation and maintenance requirements might not be suitable for those regions with limited resources [2]. Hence, natural treatment systems particularly suit to developing countries of the world.

Sustainable sanitation systems require low cost, with low energy consumption and low mechanical technology. Better choices of low cost treatment systems for rural areas are decentralized processes [3]. Treatment systems with a very small energy input, low operational cost, and low surplus sludge generation are anaerobic digesters and constructed wetlands [3–6]. Other examples of low cost natural treatment systems include oxidation ponds, anaerobic ponds, facultative ponds, terrestrial treatment systems, and vermicomposting constructed wetlands. The objective of the current review was to describe some recent advancements in the design and efficiency of various natural treatment systems and the comparison of their efficiencies. Following sections will highlight the recent developments regarding various types of natural treatment systems.

## 2. Constructed Wetlands

**Figure 1:**
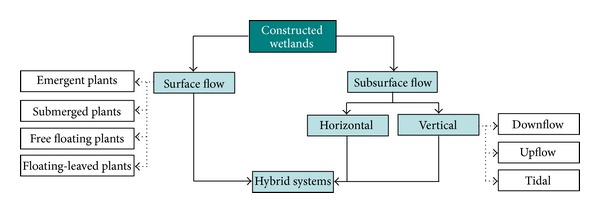
Various types of constructed wetlands [9].

Seidel [7] presented the ideas of improving inland waters suffering from high nutrients originating from sewage and sanitation through native plant species. However, at that time, experts only considered physicochemical and bacterial wastewater treatment only, and no attention was paid to controlled use of macrophytes for water purification [8].  Figure 1 shows various components of a constructed wetland [9]. Classification of constructed wetlands is based on two parameters, that is, type of macrophytic growth and water flow regime (surface and subsurface) [9]. CW can be used for the treatment of different types of wastewaters, that is, municipal, industrial, leachate, acid mine drainage, surface runoff, and so forth [10]. The emerging technology for the treatment of a variety of wastewaters is constructed wetlands (CWs) [11]. The natural wetland system uses mostly natural energy, requires low construction and operational costs, and so is energetically sustainable [12–14]. However, this assumption is not true for constructed wetlands where some energy input from human source is also required. The constructed wetlands are classified into two type, that is, free water surface (FWS) and subsurface flow (SSF) systems. In case of FWS systems, plants are rooted in the sediment layer, and water flow is above ground (surface flow). In SSF systems, plants are rooted in a porous media such as gravels or aggregates through which water flows and treatment are accomplished. SSF systems are further divided into two types: horizontal flow SSF (HSSF) and vertical flow SSF (VSSF). Compared to HSSF, the subsurface vertical flow constructed wetland (SVFCW) system is more effective for the mineralization of biodegradable organic matter and has greater oxygen transport ability [15]. For the removal of suspended solids, carbon and nitrification process vertical flow CW is more efficient, because of aerobic conditions and denitrification is poor. In VSSF CWs, feeding is intermittent (discontinuous), and the flow of wastewater is vertically administered through a substrate layer which mainly consists of sand, gravel, or a mixture of all these components [16].

Constructed wetlands can be used as an accepted eco-technology, in small towns or industries that cannot afford conventional treatment systems [17–19]. In the free water surface (FWS) type of wetland, the water is filtered through a dense stand of aquatic plants as it flows over the bed surface [20–22]. Another constructed wetland system, known as the subsurface flow wetland consists of a lined shallow basin with a gravel media and emergent aquatic plants [23–28]. Worldwide, now thousands of constructed wetlands are in use which are receiving and treating a variety of municipal, industrial, and other wastewaters [29–31]. Due to operational simplicity and cost efficiency, the utilization of constructed wetlands in Taiwan is gaining greater popularity [12, 22, 29, 32, 33]. The constructed wetland technology is now well established, but its use for treating specific industrial effluents has not been well documented [34–36]. Various kinds of constructed wetlands have been shown in Figure 1.

The technology which uses plants for the removal of contaminants from a specified area is known as green technology [37], and the process is known as phytoremediation. Phytoextraction, phytovolatilization, rhizospheric degradation, phytodegradation, and hydraulic control are the five mechanisms involved in the phytoremediation for the removal of pollutants. Different types of pollutants can be removed by phytoremediation such as heavy metals, pesticides, petroleum hydrocarbons, explosives, radionuclides, and CVOCs [38]. Heavy metals are the chemical substances whose densities are greater than 5 g cm^3^ [39]; metals cleanup requires their immobilization and toxicity reduction or removal, and they cannot be degraded like organic compounds [40]. Sedimentation/coagulation, filtration, plant uptake/removal efficiency, adsorption (binding to sand particles and root), formation of solid compounds, cation exchange, and microbial-mediated reactions, especially oxidation, are the different processes through which different types of metals can be removed in the constructed wet lands [41].

### 2.1. The Components of CW

 The basic components employed in the construction of CW are containers, plant species, and sand and gravel media in certain ratios. Other invertebrates and microbes develop naturally [42]. Combination of anaerobic reactors, vegetated reactors (CW), and natural wetlands forms ecological treatment systems. In ecologically engineered systems, different functions are performed by different communities of flora, fauna, minerals, and microbes [43]. Another important component of ecological treatment systems is microbes which carry out the different important processes like hydrolysis, mineralization, nitrification, and denitrification. Plants are critical as an attachment surface for microbes [43]. For the construction of constructed wetland, three forms of macrophytes are basically used:

1. floating macrophytes (i.e., *Lemna *spp. or *Eichornia crassipes*),
2. submerged macrophytes (i.e., *Elodea canadiensis*),
3. rooted emergent macrophytes (i.e., *Phragmites australis*, *Typha *spp.).

The plant used for phytoremediation should have high biomass, high growth rate, and ability to accumulate the target metal in the above-ground parts. They should be able to tolerate high metal concentration and have tolerance for several metals simultaneously [44].  Table 1 describes desirable features of plant species to be used in constructed wetlands.

### 2.2. Removal of Organic Matter in CW

In case of removing organic compounds by wetland plants, the focus is generally on three types of compounds, that is, chlorinated solvents, petroleum hydrocarbons, and explosives. But researchers also addressed the potential of plant species to treat other organic contaminants, such as polycyclic aromatic hydrocarbons (PAHs) and polychlorinated biphenyls (PCB) [45–47]. For wastewater purification, different natural systems involving plants, such as facultative ponds [48–50], terrestrial systems [51–55], and wetlands [8, 56–58], have been used. Floating aquatic plant systems usually contain floating macrophytes. The extensive root systems of floating leaved plants have large surface area, and rhizoplane provides an excellent site for the adhesion of rhizobacteria. In such treatment processes, rhizobacteria play an important role in the pollutant degradation and uptake [8]. A wealth of the literature has been published on the use of emergent plants [59, 60] and floating plants such as *Elodea canadensis* [61]. Although, water hyacinth is very efficient in nutrient uptake, the removal of naphthalene at toxic concentrations by *Eichhornia crassipes* has not yet been determined. *E. crassipes* is highly effective for the remediation of municipal sewage for a retention time of 2 to 5 days [62–64].

The natural systems containing various plant species were regarded as very effective and inexpensive technology for the cleanup of hazardous waste sites polluted with hydrocarbons, metals, pesticides, and chlorinated solvents [65–67]. The treatment of organic pollutants by plants may involve four mechanisms:

1. direct uptake, accumulation, and metabolism of contaminants, in plant tissues (detoxification),
2. transpiration of volatile organic hydrocarbons from leaves (avoidance),
3. release of exudates from the roots that will stimulate microbial activity and biochemical transformations (chelation), and finally,
4. the presence of mycorrhizal fungi and microbial consortia associated with the root surfaces can enhance the mineralization of pollutants in rhizosphere [68].

Photosynthetic activity and growth rate of plants are the two factors which render economic success of the treatment process by plants. Due to fast growth and large biomass production, water hyacinth (*Eichhornia crassipes* (Mart.) Solms) is largely used for the wastewater treatment [69]. Water hyacinth through uptake and accumulation can effectively remove inorganic contaminants such as nitrate, ammonium and soluble phosphorus [70, 71], and heavy metals [72, 73]. Different organic pollutants such as phenols can also be absorbed [74], but their removal mechanisms were rarely studied to confirm that the removal of these pollutants involved uptake or the enhancement of mineralization by microbial consortia associated with the root surface. 

A system comprising two parallel horizontal subsurface-flow constructed wetlands following an upflow anaerobic sludge blanket (UASB) reactor, treating municipal wastewater from the city of Belo Horizonte, Brazil, served as the basis for the evaluation of simple first-order kinetic performance models [75]. One unit was planted (*Typha latifolia*), and the other was unplanted. Tracer (Br^−^) studies were undertaken, and samples of filtered COD were collected from the inlet, outlet, and three intermediate points along the longitudinal length of the units. The following kinetic models were used, and the related reaction coefficients were calculated: (i) plug-flow model; (ii) dispersed-flow model; and (iii) tanks-in-series model. For the three models, the following variants were analysed: without and with residual COD and without and with taking into account water losses due to evapotranspiration. In general, the dispersed-flow model and the tanks-in-series model, both adjusted for residual COD and incorporating water losses, were able to give the best predictions and led to the same reaction coefficients, which were also likely to best represent the actual first-order kinetic coefficients [75].

### 2.3. Removal of Inorganics and Metals by CW

Since 1990s, CWs have been used for the treatment of wastewater to remove solids, N, P, heavy metals, and organic pollutants [76–79]. CW have also been used for the removal of coliforms from storm water, municipal sewage, and agricultural runoff [80–83]. The presence of angiospermic plants in a wetland ecosystem improves treatment efficiencies [84–86]. The unique wetlands along the coastline of tropical and subtropical regions are mangroves. Mangroves could be used in CWs for wastewater treatment as shown by different studies [81, 87, 88].

The rapid increase of shortages in resources of chemical elements (and ores) used for an increasing industrial production raises the question of alternative strategies for their acquisition [89]. Simultaneously, the elemental load in aquatic ecosystems increases by anthropogenic activities. Polluted waters are purged actively by technical treatment plants or passively by wetlands. Wetlands are known to eliminate/fix pollutants with a potentially high efficiency. Regarding this elimination/fixation potential less is known about different types of wetlands for elemental recovery. This paucity of information prompted to us to assess the impact of main processes in different types of wetlands on the recovery potential of chemical elements showing advantages and disadvantages of autochthonous and allochthonous wetlands and possible solutions. We show that autochthonous as well as allochthonous wetlands are able to accumulate high amounts of elements, but it is suggested that combining autochthonous/allochthonous processes should result in a higher efficiency [90].

The recent applications and trend of CWs in China were reviewed by Hench et al. [81] and presented the status quo, prospect, and influencing factors in CWs construction, technology application, and operation management in China based on the available data. The results of the systematic survey showed that CWs technology achieved gradual perfection under the pushes of national policies, market demand, and technical feasibility, with the capacity of wastewater treatment increasing year by year. However, there were still some problems concerning engineering operation and management. Moreover, the results demonstrated that limited by the economic level, the degree of industrialization and urbanization, climatic conditions, and land availability, CWs were distributing predominately in the region of 20°13′N–35°20′N in China, which covered the central areas with a subtropical monsoon climate and southern or central areas at province level. In these areas, there were more than 40 plant species, which accounted for 57.14% of the total number of common wetland plants. Most of the CWs composted series or parallel combination forms of the vertical flow and free water surface flow CWs units, and they were suitable to treat more than 20 different types of wastewaters. For these CWs, the effluent COD, biological oxygen demand BOD, TN, and total phosphorous (TP) reached in the ranges of 20–60 mg/L, 4–20 mg/L, 1–20 mg/L, and 0.2–1 mg/L, respectively. The effluents from CWs were reused in more than eight ways, such as for agricultural irrigation, supplying surface water, and green belt sprinkling [81].

Galvão and Matos [91] evaluated the buffering capacity of constructed wetlands by analyzing the response to sudden organic load changes. Nine horizontal flow experimental beds were divided into three equal groups and monitored in a three-phase experiment. Influent COD mass loads during Phase I were 11.4, 5.3, and 0 g/m^2^/day for Groups A–C, respectively. During Phase II there was a rise in COD mass load, and Phase III restored initial conditions. Group A showed a reduction in COD removal efficiencies with mass load increase despite more intense microbiological activity. In Group B planted beds there was a reduction of the mass load removal, which could have been due to a biofilm disturbance caused by an invasive millipede species. When mass load was lowered, removal efficiencies improved. Group C showed residual effluent COD during Phase I but provided adequate removal efficiencies (75–84%) when supplied with 1.7 g/m^2^/day during Phase II. The results of this study suggest that the buffering capacity of constructed wetlands may not be enough to maintain COD removal efficiencies during a sudden organic load increase [91].

Three free water surface constructed wetlands (FWSCWs) were built in the border of the Lake *L'Albufera de Valencia* (Valencia, Spain) [92]. The lake is the emblematic element of one of the most important wetlands in Spain, *L'Albufera de Valencia Natural Park*, and it is highly eutrophicated. The function of the set of CWs (9 ha) is treating the eutrophic water from the lake with the objective of reducing the phytoplankton population and nutrients. The treatment wetlands named as *FG* and *fp* are comprised of three basins in a series, while the last, *F4*, consists of a single cell. During the first 2 years of operation, the inflow from lake was gradually increased from 0.01 m^3^/s (April 2009) to 0.13 m^3^/s (December 2010) with the goal of establishing the maximum hydraulic loading rate (HLR) and finding the highest removal efficiency. Input concentrations of different water quality variables studied showed a high variability. The inflow contained 8.80–94 mg/L TSS, 0.16–1.13 mg/L TP, 1–17.30 mg/L TN, 0.13–13.10 mg N/L DIN, 0.10–11.50 mg NO_3_-N/L, and 3.34–257.03 *μ*g/L Chl *a*. The best removal results for these parameters were obtained in the *FG* wetland, where the average mass removal efficiencies were 75% TSS, 65% TP, 52% TN, 61% DIN, 58% NO_3_-N, and 46% Chl *a*. In the set of constructed wetlands the removal rates increased with the hydraulic load rate for TSS and TP but neither for nitrogen species nor Chl *a*; for these variables the input concentrations are a key factor in removal. For instance, mean removal rate for nitrate is 101 mg N m^−2^ d^−1^, but values higher than 400 mg N/m^2^ d were obtained when input concentrations were about 7 mg NO_3_-N/L. Values of first-order constant aerial rate (*k*_*A*_) have been obtained for all variables. The corresponding values for nitrogen species and total phosphorus are higher than obtained in previous studies, but the *k*_*A*_ value for TSS is low (94.9 m/year) owing to the eutrophic characteristics of water, and a *k*_*A*_ value for phytoplankton-Chl *a* of 65.1 m/year is introduced. The management of CWs implies the harvest of vegetation, not for removing nitrogen because nitrification-denitrification processes reduce the 83.3% of TN that enters, but for the phosphorus, limiting nutrient in the *Lake L'Albufera*, that now is accumulated in plants and soils but could be sent back in several years [92].

In 2008, concentrations of iron and manganese in the sediments of seven constructed wetlands (CWs) with horizontal subsurface flow in the Czech Republic were evaluated [93]. The surveyed constructed wetlands varied in the length of operation between 2 and 16 years at the time of sampling. In each constructed wetland three samples of sediment were taken in the inflow, middle, and outflow zones to the depth of 0.6 m. The sample was divided into top (0–20 cm) and bottom (20–60 cm) sections, resulting in a total number of 18 samples in each system. In each sample the amount of sediment on dry mass basis was evaluated and concentrations of Fe and Mn in the sediment were determined using ICP-MS. The survey revealed that the amount of sediments in the filtration bed increases with the length of operation. In general, greater sediments were located at the bottom layers due to the wastewater distribution near the bottom. Concentrations of manganese in the sediment were highest in the new systems and decreased with length of operation. With the exception of one CW, in all other constructed wetlands Mn concentration in the sediment was significantly higher within the top layer. Mn concentrations in sediments found in that study were found within the concentration range commonly occurring in natural unpolluted wetlands. Iron concentration in the sediment also decreased with the increasing length of operation, but the dependence on the operation time was not as clear as for manganese. The concentration of iron was comparable with other studies from constructed wetlands treating sewage but higher as compared to polluted sediments which are usually highly anaerobic. The results clearly demonstrated that in order to evaluate the amount of iron and manganese in the filtration beds it is necessary to take into consideration both concentration of the elements and the amount of sediment accumulated in the filtration bed. Despite the lowest concentrations in the sediment the highest accumulated amount of both iron and manganese was found in the oldest CW Spálené Poříčí because of the highest concentration of sediment in the filtration bed [94]. 

In another investigation by Paulo et al. [93], the blackwater fraction was treated in an evapotranspiration tank (TEvap) system, whereas the greywater fraction was treated by a compact setup including a grease trap, sedimentation tank, and two constructed wetlands. Results of both systems, obtained during a 400-day trial in a 9-person household in Campo Grande-MS, Brazil, showed that ecological system could be an economical alternative to conventional septic tank solutions. The treatment system managed both greywater and blackwater at household level, enabling the development of green areas, improving microclimate and allowing for the reuse of grey water and the nutrients present in black water. The TEvap system was essentially maintenance free, but the constructed wetlands did require attention, to prevent clogging of subsystems [93].

### 2.4. Role of Rhizosphere in Pollutant Uptake

The term “rhizosphere” was introduced by the German scientist Hiltner [95]. Rhizosphere is a border line between soil and plants, which plays an important role in the agroenvironmental structure [96], where physiochemical and biological characteristics of soil, biomass activity, and community structure of microorganisms are significantly affecting each other [97]. The rhizosphere is an environment around plants where pathogenic and beneficial microorganisms create a potential strength on plant growth and health [98]. The rhizosphere is usually occupied by microbial groups like bacteria, fungi, nematodes, protozoa, algae, and microarthropods [98, 99]. Proper understanding of the ecosystem is necessary for the waste treatment through beneficial integration of microorganisms and suitable selection of microbes [100].

It has been proved that plant roots attract soil microorganisms through their exudates which ultimately results in variations of the rates of metabolic activity of the rhizosphere microbial communities and those of the nonrhizosphere soil [101]. Generally, the plants associated bacteria migrate from the soil to the rhizosphere of living plant and ultimately colonize the rhizosphere and roots of plants [102]. These rhizobacteria are symbiotic partners of plants and are considered as plants growth promoting microbes [103]. Due to natural activities of colonizing, bacterial species along the surface of the roots of the inoculated plants enhance the growth rate of plants [104].

The exudates released by plant roots and associated microbes may significantly mobilize heavy metals and ultimately increase their bioavailability [105]. Bacteria are the most common type of soil microorganisms due to their rapid growth and utilization of wide range of substances like carbon or nitrogen sources. Rhizospheric microbes may play a dual role in soil ecological potential both constructively as well as destructively. The number and variety of harmful and useful microorganisms are related to the quality and quantity of rhizodeposits and the effect of the microbial relations that occurs in the rhizosphere [106]. 

An alternate way for the remediation of heavy metals toxicity is the use of rhizospheric microorganisms [107]. A variety of useful free-living soil bacteria are usually referred to as plant growth-promoting rhizobacteria and are found in association with the roots of diverse plants species [108]. Plants and microbes possess a strong and valuable relationship but may have strong competition for resources, including nutrients and water [109]. In both natural and manmade ecosystems, plant associated bacteria play a key role in host adaptation to a changing environment. These microorganisms can modify plant metabolism, so that upon exposure to heavy metal stress, the plants are able to tolerate their high concentrations [110]. Several authors have pointed out bacterial biosorption or bioaccumulation mechanism and other plant growth promoting factors; the production of ACC deaminase and phytohormones were regarded as key for better plant growth in heavy metals contaminated soils [111–113]. *Sedum alfredii*, a terrestrial Zn/Cd hyperaccumuluator, of Zn, Cd, Cu, and Pb from contaminated water evidently improved the phytoremediation in the presence of naturally occurring rhizospheric bacteria, also by using antibiotic ampicillin [114].

### 2.5. Vermicomposting and Constructed Wetlands

Vermicomposting principally employs earthworms to ingest organic matter and consequently egests a nutrient-rich cast that can be used as a soil conditioner. After fragmentation and ingestion, the microbial activity for the decomposition process is enhanced [115]. The process was also studied in relation to the changes in the composition properties of the wastes [116]. The advantages of this technology include speeding up and assisting the composting process as well as the quality of the end products. In addition, vermicomposting is considered to be odor-free because earthworms release coelomic fluids in the decaying waste biomass which have antibacterial properties [117]. Pathogens are also killed according to this effect. Many workers have highlighted the great reduction of pathogenic microorganisms by vermicomposting [118–120]. Among vermicomposters, earthworms are the most important invertebrates which play a significant role in the degradation of organic matters to humus. Earthworms can be classified as detritivores and geophages according to their feeding habit [121]. Detritivores feed on plant litter or dead roots and other plant debris as well as on mammalian dung. These earthworms are called humus formers, and they include the epigeic and anecic earthworms like *Perionyx excavatus*, *Eisenia fetida*, *Eudrilus euginae*, and *Polypheretima elongate* [122]. Geophagous worms mostly influence the aeration and mixing of subsoil; that is why they are called endogeic earthworms. Both types have their role, either as composters of detritivores or fieldworkers for geophages [123].

For using earthworms in constructed wetlands, it is imperative to understand their growth conditions. Species such as *Perionyx excavatus* and *Eudrilus eugeniae* are more common in warmer climates. They would be more suitable for the vermicomposting process in those regions. For instance, in Africa it is recommended to use *Eudrilus eugeniae*, which can reach sexual maturity in as little as five weeks compared with *E. fetida *which requires 6–8 weeks [124] and *Perionyx excavatus* in Asia as they are widely distributed. Both of these species are most productive at 25°C, which is higher than the optimal temperature quoted for other species in temperate regions [125]. In Thailand, the species commonly used is *Pheretima peguana*. The tolerance under different temperatures varies considerably for each species, whereas their optimum moisture requirements, C : N ratio, and ammonia content do not vary greatly [119]. The temperature tolerance for some species as well as their distribution is described and compared in Table 2.

In principle, earthworms prefer an aerobic condition [119]. Therefore, this should be applicable for the VSFCWs due to intermittent feeding rather than the anaerobically-operated HSFCWs. VSFCWs could offer a viable habitat for earthworm populations because of their ability to transfer oxygen to the root zone. This ability creates the aerobic micro-sites within the largely anoxic environment [126]. Under anoxic conditions, the earthworms will die. Edwards [119] has described some optimal conditions for breeding earthworms (*E. fetida*) in animal and vegetable wastes. However, future researches should be focused on the performance of earthworm based vermicomposting CW which are fed by various kinds of wastewaters like domestic, municipal, and some selected industrial wastewaters containing inorganic toxic pollutants like ammonia, heavy metals, and low DO. Nuengjamnong et al. [127] treated swine wastewater by integrating earthworms into constructed wetlands. They investigated the application of integrating earthworms (*Pheretima peguana*) into two-stage pilot-scale subsurface-flow constructed wetlands (SFCWs) receiving swine wastewater in terms of their treatment performance, namely, organic content, total Kjeldahl nitrogen (TKN), and solid reduction as well as the quantity of sludge production. There was a minor difference in terms of removal efficiency according to each parameter when comparing the unit with earthworms to the one without earthworms. Both achieved the TKN, BOD, COD, total volatile suspended solids (TVSS), suspended solids (SS), and total solids (TS) removal by more than 90%. The earthworms helped in reducing the sludge production on the surface of constructed wetlands 40% by volume, which resulted in lowering operational costs required to empty and treat the sludge. The plant biomass production was higher in the wetlands without earthworms. Further research could be undertaken in order to effectively apply earthworms inside the wetlands [127]. 

Enhancement of rural domestic sewage treatment performance and assessment of microbial community diversity and structure using tower vermifiltration was investigated by Wang et al. [128]. The performance of a novel three-stage vermifiltration (VF) system using the earthworm, *Eisenia fetida*, for rural domestic wastewater treatment was studied during a 131-day period. The average removal efficiencies of the tower VF planted with *Penstemon campanulatus* were as follows: COD, 81.3%; ammonium, 98%; total nitrogen, 60.2%; total phosphorus, 98.4%; total nitrogen, mainly in the form of nitrate. Soils played an important role in removing the organic matter. The three-sectional design with increasing oxygen demand concentration in the effluents and the distribution of certain oxides in the padding were likely beneficial for ammonium and phosphorus removal, respectively. The microbial community profiles revealed that band patterns varied more or less in various matrices of each stage at different sampling times, while the presence of earthworms intensified the bacterial diversity in soils. Retrieved sequences recovered from the media in VF primarily belonged to unknown bacterium and *Bacilli of Firmicutes* [128]. Wang et al. [129] investigated the impact of fly ash and phosphatic rock on metal stabilization and bioavailability during sewage sludge vermicomposting. Sewage sludge (SS) was mixed with different proportions of fly ash (FA) and phosphoric rock (PR), as passivators, and earthworms, *Eisenia fetida*, were introduced to allow vermicomposting. The earthworm growth rates, reproduction rates, and metal (except Zn and Cd) concentrations were significantly higher in the vermireactors containing FA and PR than in the treatments without passivators. The total organic carbon (TOC) and total metal concentrations in the mixtures decreased, and the mixtures were brought to approximately pH 7 during vermicomposting. There were significant differences in the decreases in the metal bioavailability factors (BFs) between the passivator and control treatments, and adding 20% FA (for Cu and Zn) or 20% PR (for Pb, Cd, and As) to the vermicompost were the most effective treatments for mitigating metal toxicity. The BF appeared to be dependent on TOC in the all treatments but was not closely dependent on pH in the different vermibeds [129].

### 2.6. Role of Mangroves

Mangroves are sole wetlands along the coastline of tropical and subtropical areas. They have unique adaptations to stressed environments and a massive requirement for nutrients because of fast growth, high primary productivity, metabolism, and yield. Studies suggested that mangroves could be used in CWs for wastewater treatment [81, 88, 130]. Studies were focused on the treatment efficiency of the mangrove species *Kandelia candel*. Little work has been done on the assessment of nutrient removal efficiency of different mangrove species [131]. They have special adaptations to stressful saline environments and a huge demand for nutrients because of rapid growth, high primary productivity, metabolism, and turnover. Many studies have demonstrated that mangroves could be used in CWs for wastewater treatment [81, 88, 130]. Plants can affect their growth medium by excreting exogenous enzymes and can also affect microbial species composition and diversity by releasing oxygen into the rhizosphere that in turn indirectly influences enzyme activity [132, 133]. Nevertheless, the relationship between plant species composition and enzyme activities in CW systems is poorly understood. The subsurface vertical flow constructed wetland (SVFCW) system with unsaturated flow possesses greater oxygen transport ability than the horizontal subsurface flow beds and is more effective for the mineralization of biodegradable organic matter.

The use of a mangrove plantation as a constructed wetland for municipal wastewater treatment was studied by Boonsong et al. [89]. The study evaluated the possibility of using mangrove plantation to treat municipal wastewater. Two types of pilot scale (100 × 150 m^2^) free water surface constructed wetlands were set up at the Royal Laem Phak Bia Environmental Research and Development Project in central Thailand. One system is a natural *Avicennia marina* dominated forest system. The other system is a new mangrove plantation system in which seedlings of *Rhizophora* spp., *A. marina*, *Bruguiera cylindrical,* and *Ceriops tagal* were planted at 1.5 × 1.5 mintervals, making up 4 strips of 37.5 × 100 m^2^ each. Wastewater from municipal and nearby areas was collected and pumped into the systems then retained within the systems for 7 and 3 days, respectively, before discharging. The results indicated that the average removal percentage of TSS, BOD, NO_3_-N, NH_4_-N, TN, PO_4_-P, and TP in the new-plantation systems were 27.6–77.1, 43.9–53.9, 37.6–47.5, 81.1–85.9, 44.8–54.4, 24.7–76.8, and 22.6–65.3, respectively, whereas the removal percentage of those parameters in the natural forest system were 17.1–65.9, 49.5–51.1, 44.0–60.9, 51.1–83.5, 43.4–50.4, 28.7–58.9, and 28.3–48.0, respectively. Generally, the removal percentage within the new-plantation system and the natural forest system was not significantly different. However, when the removal percentages with detention time were compared, TSS, PO_4_-P, and TP removed percentages were significantly higher in the 7-day detention time treatment. Even with the highly varied and temporally dependent percentage removal of TSS, BOD, and nutrients, the overall results showed that a mangrove plantation could be used as a constructed wetland for municipal wastewater treatment in a similar way to the natural mangrove system. Therefore, the use of mangrove plantations for municipal wastewater treatment is applicable [89].

A pilot-scale mangrove wetland was constructed in Futian (China) for municipal sewage treatment by Yang et al. [134]. Three identical belts (length: 33 m, width: 3 m, and depth: 0.5 m) were filled with stone (bottom), gravel, and mangrove sand (surface). Seedlings of two native mangrove species (*Kandelia candel, Aegiceras corniculatum*) and one exotic species (*Sonneratia caseolaris*) were transplanted to the belts with one species for each belt. The hydraulic loading was 5 m^3^d^−1^ and hydraulic retention time 3 d. High levels of removal of COD, BOD(5), TN, TP, and NH_3_-N were obtained. The treatment efficiency of *S. caseolaris* and *A. corniculatum* was higher than that of *K. candel*. Faster plant growth was obtained for *S. caseolaris*. The substrate in the *S. caseolaris* belt also showed higher enzyme activities including dehydrogenase, cellulase, phosphatase, urease, and beta-glucosidase. The removal rates of organic matter and nutrients were positively correlated with plant growth. The results indicated that mangroves could be used in a constructed wetland for municipal sewage treatment, providing that posttreatment to remove coliforms was also included [134]. Saline municipal wastewater treatment was investigated in constructed mangrove wetland by Li et al. [135]. The feasibility of using constructed mangrove wetlands to treat saline municipal wastewater was evaluated in the study. Constructed wetland, acting as an ecological engineering alternative, is capable of reducing NH_4_^+^-N TN TP and COD from saline wastewater. During the 10 months' operating period, the constructed wetland was operated with salinity increasing from 10 to 50 parts per thousands (ppt). When the salinity of wastewater was below 30 ppt, the removal rates of NH_4_^+^-N>, TN>, TP, and COD were 71.6 79.8%, 75.5–89.6%, 82.7–97.4, and 66.6–85.3%, respectively. The removal rate of COD decreased to about 40% when the salinity increased to 50 ppt. A good performance of COD removal was obtained when the constructed mangrove wetland was operated at loading rate between 12.6–18.9 g m^−2^ d^−1^. The preliminary result showed that constructed mangrove wetland for saline municipal wastewater treatment had a broad future [135].

## 3. Land Treatment Systems

Terrestrial or land treatment systems utilize land to treat wastewater. The land is generally allowed to flood with wastewater to be treated; the extent and treatment conditions usually depend on many factors like soil characteristics, the characteristics of wastewater, topography, the presence of additional media in soil, and so forth. When these systems are used, large buffer areas and fencing may be required to ensure minimal human exposure [136]. Also, given the nature of these systems, all requirements include disinfection and significant pretreatment before application. In wet and cold areas, an additional basin for storage or a larger dosing tank is necessary to eliminate possible runoff from the application area. The most used variation of these systems is the spray irrigation system. Spray irrigation systems distribute wastewater evenly on a vegetated plot for final treatment and discharge. Spray irrigation can be useful in areas where conventional onsite wastewater systems are unsuitable due to low soil permeability, shallow water depth table or impermeable layer, or complex site topography. Spray irrigation is not often used for residential onsite systems because of its large areal demands, the need to discontinue spraying during extended periods of cold weather, and the high potential for human contact with the wastewater during spraying. Spray irrigation systems are among the most land-intensive disposal systems. Buffer zones for residential systems must often be as large as, or even larger than, the spray field itself to minimize problems [137].

In spray irrigation system, pretreatment of the wastewater is normally provided by a septic tank (primary clarifier) and aerobic unit, as well as a sand (media) filter and disinfection unit. The pretreated wastewater in spray irrigation systems is applied at low rates to grassy or wooded areas. Vegetation and soil microorganisms metabolize most nutrients and organic compounds in the wastewater during percolation through the first several inches of soil. The cleaned water is then absorbed by deep-rooted vegetation, or it passes through the soil to the ground water [136].

Rapid infiltration (RI) is a soil-based treatment method in which pretreated (clarified) wastewater is applied intermittently to a shallow earthen basin with exposed soil surfaces. It is only used where permeable soils are available. Because loading rates are high, most wastewater infiltrates the subsoil with minimal losses to evaporation. Treatment occurs within the soil before the wastewater reaches the ground water. The RI alternative is rarely used for onsite wastewater management. It is more widely used as a small-community wastewater treatment system in the United States and around the world [136].

The third and last type of land surface treatment is the overland flow (OF) process. In this system, pretreated wastewater is spread along a contour at the top of a gently sloping site that has minimum permeability. The wastewater then flows down the slope and is treated by microorganisms attached to vegetation as it travels by sheet flow over very impermeable soils until it is collected at the bottom of the slope for discharge. This system requires land areas similar to the spray irrigation system. However, surface water discharge requirements (e.g., disinfection) from the OF system must still be met. Overland flow, like rapid infiltration, is rarely used for onsite wastewater management [136].

Land treatment systems comprise a possible alternative solution for wastewater management in cases where the construction of conventional (mechanical) wastewater treatment plants (WWTPs) are not afforded or other disposal option are not accessible. They have established to be an ideal technology for small rural communities, homes, and small industrial units due to low energy, low operational, and maintenance costs [137]. They depend upon physical, chemical, and biological reactions on and within the soil. Slow rate OF systems require vegetation, both to take up nutrients and other contaminants and to slow the passage of the effluent across the land surface to ensure the maximum contact times between the effluents and the plants/soils. Slow rate subsurface infiltration systems and RI systems are “zero discharge” systems that rarely discharge effluents directly to streams or other surface waters. Each system has different constraints regarding soil permeability. Slow rate OF systems are the most expensive among all the natural systems to put into practice. 

The OF systems are a land application treatment technique in which treated effluents are ultimately discharged to surface water. The major profits of these systems are their low maintenance and technical manpower requirements. Subsurface infiltration systems are designed for municipalities of less than 2,500 people. They are usually designed for individual homes (septic tanks), but they can be designed for clusters of homes. Although they do require specific site conditions, they can be low-cost methods of wastewater disposal.

The use of subsurface infiltration systems has been expanded to treat various types of wastewater, including landfill leachates [138], dairy effluents [139], meat processing wastewater [140], olive oil mill wastewater [141], agricultural drainage [142], and contaminated groundwater [143]. Recognizing the importance of wastewater management in meeting future water demands, preventing environmental degradation, and ensuring sustainable growth, the use of subsurface infiltration systems in wastewater management is expected to increase.

### 3.1. Fundamental Processes

Slow rate systems purify the applied wastewater through physical, chemical, and biological mechanisms that occur concurrently in the soil, water, and atmosphere environment. These mechanisms include filtration, transformation, degradation, predation, natural die off, soil adsorption, chemical precipitation, denitrification, volatilization, and plant uptake. Detailed knowledge of the factors regulating these fundamental processes, as well as the complex interactions among them, is prerequisite for achieving a reliable treatment, particularly in terms of organic matter degradation, pathogen elimination, and nutrient removal. Moreover, the long-term treatment performance and the sustainability of the land remain equally important issues. Plant selection is among the most critical components mediating the successful performance of SRS [144]. Vegetation may dramatically affect the performance of land treatment systems through its effects on hydraulic loading, nutrient uptake, biomass production, microbial community (structure and activity), and other particular functions such as trace elements uptake/inactivation and toxic organics degradation/inactivation.

### 3.2. Vegetation of Terrestrial Systems

The primary criteria for vegetation selection are (a) water requirements, (b) the potential for nutrient uptake, (c) salt tolerance, (d) trace elements uptake and/or tolerance, and (e) biomass production. 

Table 3 shows some examples of popular plant species for wastewater treatment. Further features that should be taken into consideration include climate (frosts, temperature, photoperiod, and length of growing season), soil properties (pH, salt, and nutrient concentration), plant availability, length of vegetation cycle, and production direction (e.g., pulp, wood, biofuels, and other products). Currently, a variety of annual crops, perennial grasses, and forest trees are used in slow rate systems (SRS) worldwide. Comparative accounts of various design features of all terrestrial treatment systems have been demonstrated in Table 4.

### 3.3. Soil-Aquifer Treatment (SAT)

The soil layers in an SAT system allow the recycled water to undergo further physical, biological, and chemical purification as it passes through the soil. Physical treatment takes place by the soil acting as a filter, removing particles that may still be in the water. The biological treatment takes place as the microorganisms that naturally live in the soil consume or break down the organic matter that may still be in the recycled water. Chemical treatment occurs in the soil through such natural processes as neutralization, reduction, and oxidation during which one substance or compound in the recycled water is removed, broken down, or transformed into another. Changes also take place as the recycled water reaches the groundwater aquifer and mixes with the water already there and moves through the aquifer to extraction wells [145]. There are two zones where physical, chemical, and biological purification processes take place underground. The first is in what is called the unsaturated zone. The second is the saturated zone. 

The unsaturated zone consists of the upper layers of the soil, unconsolidated sediments, and bedrock where spaces between soil particles are not completely filled with water like a kitchen sponge that is damp (i.e., not saturated). Scientists may also call this area the vadose zone or soil percolation zone. The top few inches of the soil are called the infiltration interface where the recycled water is in contact with the soils for only a few minutes. It is a very active zone of treatment. The rest of the unsaturated or soil percolation zone is typically 10 to 100 ft. deep where the recycled water is in contact with the soils ranging from several hours to days where additional purification occurs [145].

The saturated zone consists of lower layers of the soil and sediment and rock formations where all the spaces are filled with water like a kitchen sponge that is completely wet (e.g., saturated). The thickness of the saturated zone is the vertical depth or extent of an aquifer. The upper surface of the saturated zone is called the water table. After the SAT water reaches the saturated zone or aquifer, it mixes together with the underground water and then moves slowly to extraction wells. During this contact time with the aquifer material and mixing with the native groundwater, further purification of the recycled water occurs [145].

A pilot study was carried out in Sabarmati River bed at Ahmedabad, India for renovation of primary treated municipal wastewater through soil aquifer treatment (SAT) system [146]. The infrastructure for the pilot SAT system comprised of two primary settling basins, two infiltration basins, and two production wells located in the centre of infiltration basins for pumping out renovated wastewater. The performance data indicated that SAT has a very good potential for removal of organic pollutants and nutrients as well as bacteria and viruses. The SAT system was found to be more efficient and economical than the conventional wastewater treatment systems and hence recommended for adoption. A salient feature of the study was the introduction of biomat concept and its contribution in the overall treatment process [146]. SAT proved to be efficient, economical, and feasible method for wastewater treatment [147]. SAT system achieved an excellent reduction of BOD, suspended solids, and fecal coliform. About 90% of water applied to SAT site was returned to watershed. A case study was made to increase the efficiency of the system [148]. A water quality model for water reuse was made by mathematics induction [148]. The relationship among the reuse rate of treated wastewater (*R*), pollutant concentration of reused water (*C*_*s*_), pollutant concentration of influent (*C*_0_), removal efficiency of pollutant in wastewater (*E*), and the standard of reuse water were discussed in this study. According to the experiment result of a toilet wastewater treatment and reuse with membrane bioreactors, *R* would be set at less than 40%, on which all the concerned parameters could meet with the reuse water standards. To raise *R* of reuse water in the toilet, an important way was to improve color removal of the wastewater [148].

Through the use of innovative analytical tools, the removal/transformation of wastewater effluent organic matter (EfOM) has been tracked through soil aquifer treatment (SAT) [149]. While the total amount of EfOM is significantly reduced by SAT, there are trends of shorter term versus longer term removals of specific EfOM fractions. The preferential removal of nonhumic components (e.g., proteins, polysaccharides) of EfOM occurs over shorter travel times/distances, while humic components (i.e., humic substances) are removed over longer travel times/distances, with the removal of both by sustainable biodegradation. Dissolved organic nitrogen (DON), a surrogate for protein-like EfOM, is also effectively removed over shorter term SAT. There is some background humic-like natural organic matter (NOM), associated with the drinking water source within the watershed that persists through SAT. While most effluent-derived trace organic compounds are removed to varying degrees as a function of travel time and redox conditions, a few persist even through longer term SAT [149].

The design of a proper soil-aquifer treatment (SAT) groundwater recharge system is proposed after studying the geological and hydrogeological regime of the coastal aquifer system at Nea Peramos, NE Greece [150]. The investigation of the qualitative problem of the study area included groundwater level measurements and groundwater sampling and chemical analyses, respectively. The paper also includes the design of necessary maps, such as geological, piezometric, and distribution of specific qualitative parameters. The investigation concluded to further research and managerial useful proposals [150].

## 4. Aquatic Systems

Any watery environment, from small to large, from pond to ocean, in which plants and animals interact with the chemical and physical features of the environment is called an aquatic system. Since ages, the usual practice of wastewater disposal was its release into natural aquatic water bodies. Groundwater and surface water systems should be protected from organic matter, pathogens, and nutrients, coming from natural and anthropogenic resources. Both grey and black water coming from any residential units should be treated prior to its discharge to surface waters for reclamation and secondary uses so that it may not make hazards to human beings and to the environment [151]. Natural aquatic systems work on the natural ecological principals where aquatic plants, algae, and other microbes absorb pollutants found in the wastewater to accomplish treatment. Wastewater ponds are one of the convenient options for the effective pollutant removal [152]. The proceeding section has been reserved for recent advancements on the use of various ponds to treat wastewaters.

### 4.1. Design of Wastewater Pond Systems

Wastewater ponds are natural systems whose biochemical and hydrodynamic processes are influenced by meteorological factors such as sunshine, wind, temperature, rainfall, and evaporation [153]. Sun is the driving force in the purification process of pond systems as is in any natural water body. Wastewater ponds, however, differ greatly from natural water bodies, for example, lakes and oceans in nutrient loading, oxygen demand, depth, size, water residence time, material residence time, and flow pattern [154]. Variations in meteorological factors often trigger fluctuations in water quality parameters, such as, temperature, pH, and dissolved oxygen (DO) both seasonally and diurnally. Photosynthetic oxygenation, which is essential for aerobic oxidation of the waste organics, varies diurnally with light intensities with peaks occurring between 1300 and 1500 h in the tropics [155]. It is not uncommon to find dissolved oxygen supersaturation between 300% and 400% on the top water layers of algal ponds on warm sunny afternoons in the tropics [50, 155–157]. The pH of algal ponds increases with photosynthesis as algae continue consuming carbon dioxide faster than it can be produced by bacterial respiration. As CO_2_ diffusion from the atmosphere is minimal, primarily due to elevated surface water temperatures, the CO_2_ deficit during peak photosynthesis is met from the dissociation of bicarbonate ions. This bicarbonate dissociation with concomitant consumption of CO_2_ by algae increases the concentration of hydroxyl ions in the water column causing the pH to rise to well over 10 [158–160].

Temperature has a pronounced effect on both biochemical and hydrodynamic processes of pond systems. During the daylight hours, solar radiation heats up the top water layer thus causing thermal stratification with a consequence of the warmer and lighter water overlaying the cooler and denser deeper water. Such density stratification reduces the performances of pond systems by increasing short-circuiting and disrupting the internal diffusive and advective mass transfer mechanisms. Thermal stratification also localizes algae into bands of 10–15 cm width that move up and down through the water column in response to changes in light intensity [161]. Seasonal and diurnal variations of temperature, pH, and dissolved oxygen in advanced integrated wastewater pond system treating tannery effluent were studied by Tadesse et al. [161]. Seasonal and diurnal fluctuations of pH, dissolved oxygen (DO), and temperature were investigated in a pilot-scale advanced integrated wastewater pond system (AIWPS) treating tannery effluent. The AIWPS was comprised of advanced facultative pond (AFP), secondary facultative pond (SFP), and maturation pond (MP) all arranged in series. The variations of pH, DO, and temperature in the SFP and MP followed the diurnal cycle of sunlight intensity. Algal photosynthesis being dependent on sunlight radiation, its activity reached climax at early afternoons with DO saturation in the SFP and MP in excess of over 300% and pH in the range of 8.6–9.4. The SFP and MP were thermally stratified with gradients of 3–5°C/m, especially, during the time of peak photosynthesis. The thermal gradient in the AFP was moderated by convective internal currents set in motion as a result of water temperature differences between the influent wastewater and contents of the reactor. In conclusion, the AFP possessed remarkable ability to attenuate process variability with better removal efficiencies than SFP and MP. Hence its use as a lead treatment unit, in a train of ponds treating tannery wastewaters, should always be considered [161].

To improve the hydraulic characteristics of the pond system, various innovations and modifications have been made in basic designs of the pond systems in the last two decades along with the advances of efficient aeration tools. There is a wide range of the pond systems depending upon the processes, characteristics, design methods, and operating procedures, that is, complete mix ponds, complete retention pond, facultative ponds, partial-mix ponds, anaerobic ponds, high-performance aerated pond systems (complex designs), controlled discharge pond, hydrograph controlled release, proprietary in pond systems for nitrogen removal, for nitrification, and denitrification, high-performance modified aerated pond systems, phosphorus removal ponds, ponds coupled with wetlands and gravel beds system for nitrogen removal, nitrification filters and settling basins for control of algae, and hydraulic control of ponds.

### 4.2. Overview of Pond Systems for Wastewater Treatment

If inexpensive ground is available then stabilization pond is one of the frequent wastewater treatment methodologies. Usually, waste stabilization ponds (WSPs) are huge, man-made water ponds [151]. The waste stabilization pond involves the construction of an artificial pond or the setting aside of a suitable natural pond or lagoon. The liquid sewage, released into the pond either before or after preliminary treatment, is held there to permit desired microbiological transformations to take place [162]. These ponds are firstly overflowed with wastewater and then treated by means of natural processes. Separately or connected in a series for improved wastewater treatment ponds can be used. There are three types of stabilization ponds, that is, (1) anaerobic ponds, (2) facultative ponds, and (3) aerobic (maturation) ponds. All of these have different action and design distinctiveness [152]. For effective performance, WSPs should be connected in a sequence of three or more with waste matter to be transferred from the anaerobic to the facultative pond and finally to the aerobic pond. As a pretreatment phase, the anaerobic pond minimizes the suspended solids (SS) and biological oxygen demand (BOD) [163]. Where the entire deepness of the pond is anaerobic, the pond is a man-made deep lake. Anaerobic ponds are built for a depth of 2 to 5 m for little detention time of 1 to 7 days. A complete design manual must be consulted for all kinds of WSPs, and the actual design will depend on the wastewater characterization and the loading rates [152]. Organic carbon is converted into methane through anaerobic bacteria, and it also removes about 60 to 65% of the BOD. To offer a high level of pathogen removal numerous aerobic ponds can be built in series. Away from residential areas and public spaces, they are particularly suitable for rural community that has open unused lands. They are not suitable for very dense or urban areas. WSPs are mainly competent in warm, sunny climate and work in most climates. For efficient treatment, the retention times and loading rates can be adjusted in case of cold climates [151].

The primary costs associated with constructing an anaerobic pond are the cost of the land, building earthwork appurtenances, constructing the required service facilities, and excavation. Costs for forming the embankment, compacting, lining, service road and fencing, and piping and pumps must also be considered. Operating costs and energy requirements are minimal [164].

To examine and assess the possible alternatives in modern societies, environmental management programs use models of systems [151]. Waste stabilization ponds are commonly well thought-out as being efficient for intestinal parasites removal [165]. Rectangular and cross baffle shapes are popularly capable to improve water quality. To maintain the ponds formation of scum, surplus solids and compost and garbage should be avoided to enter in the ponds. To make sure that animals and people stay away from the area, a fence should be installed, and surplus trash should not enter in the ponds [166].

Aerated pond is a huge, outside, diverse aerobic reactor. Mechanical aerators are installed to give oxygen (O_2_) and to sustain the aerobic microorganisms mixed and suspended in the water body for high rate nutrient removal and organic degradation [151]. To reduce the interference with the aerators influent must be subjected to screening and pretreatment to remove trash fine and coarse particles [152]. Increased mixing along aeration through mechanical components in aerated ponds ensures high organic loading in the ponds. This results the increased organic matter degradation and pathogen removal. In colder climates, aerated ponds can sustain because of the continuous supply of oxygen by the mechanical units besides the light-induced photosynthesis [163]. A settling tank is necessary to separate the effluent from the solids, since the exposure to air causes turbulence. They are mainly suitable for the areas of reasonably priced lands that are away from residences settlements and businesses communities [151].

The advantages include reliable BOD_5_ removal; significant nitrification of NH_3_ possible with sufficient mean cell resident time; treatment of influent with higher BOD_5_ in less space; and reduced potential for unpleasant odors. Aerated ponds are more complicated to design and construct, which increases capital and O&M costs. A larger staff is needed for whom training must be provided on a regular basis. Finally, sludge removal is more frequent and requires secondary treatment for disposal off-site [164].

Construction costs associated with partially mixed aerobic ponds include cost of the land, excavation, and inlet and outlet structures. If the soil where the system is constructed is permeable, there will be an additional cost for lining. Excavation costs vary, depending on whether soil must be added or removed. Operating costs of partial mix ponds include operation and maintenance of surface or diffused aeration equipment [164].

Facultative ponds have been in use for more than 100 years in USA [164]. These ponds are usually 1.2–2.4 m in depth and are not mechanically mixed or aerated. The layer of water near the surface contains sufficient DO from atmospheric reaeration and photosynthetic oxygenation by microalgae growing in the photic zone to support the growth of aerobic and facultative bacteria that oxidize and stabilize wastewater organics. The bottom layer of a conventional facultative pond includes sludge deposits that are decomposed by anaerobic bacteria. These shallow ponds tend to integrate carbon and primary solids undergoing acetogenic fermentation but only intermittent methane fermentation. The intermediate anoxic layer, called the facultative zone, ranges from aerobic near the top to anaerobic at the bottom. These three strata or layers may remain stable for months due to temperature-induced water density differentials, but normally twice a year during the spring and fall seasons, conventional facultative ponds will overturn, and the three strata will mix bottom to top, top to bottom. This dimictic overturn inhibits CH_4_ fermentation by O_2_ intrusion into the bottom anaerobic stratum, and, as a result, C is integrated rather than being converted into biogas [167]. The presence of algae, which release O_2_ as they disassociate water molecules photo-chemically to assimilate hydrogen during photosynthesis, is essential to the successful performance of conventional, as well as advanced, facultative ponds [164]. 

The advantages of facultative ponds include infrequent need for sludge removal, effective removal of settle-able solids, BOD_5_, pathogens, fecal coliform, and, to a limited extent, NH_3_. They are easy to operate and require little energy, particularly if designed to operate with gravity flow. The disadvantages include higher sludge accumulation in shallow ponds or in cold climates and variable seasonal NH_3_ levels in the effluent. Emergent vegetation must be controlled to avoid creating breeding areas for mosquitoes and other vectors. Shallow ponds require relatively large areas. During spring and fall dimictic turnover, odors can be an intermittent problem [164].

### 4.3. Energy Requirements for Various Natural Treatment Systems

The available cost data for different types of natural treatments systems are difficult to interpret given the number of design constraints placed on the various systems. The size required and resulting costs will vary depending upon whether the systems are designed to remove BOD_5_, TSS, NH_3_, or total N. Various reports by different authors have highlighted the costs involved in the construction and operation of these systems which include EPA [164], Crites and Fehrmann [168] and Shilton [169]. Energy consumption is a major factor in the operation of wastewater treatment facilities. As wastewater treatment facilities are built to incorporate current treatment technology and to meet regulatory performance standards, the cost of the energy to run the processes must be considered more carefully in the designing and planning of the facilities. Planners and designers should seek out the most recent information on energy requirements so as to develop a system that incorporates the most efficient and affordable type and use of energy to treat wastewater to meet regulatory requirements consistently and reliably [164]. Expected energy requirements for various wastewater treatment processes are shown in Table 5. Facultative ponds and land application processes can produce excellent quality effluent with smaller energy budgets.

## 5. Conclusions

Currently, economic crunch in many developed as well as developing nations is forcing to implement low-cost natural treatment systems for the domestic and industrial wastewater treatment. In case the technological treatment facilities are installed in many developing countries, the energy input is difficult to be supplied in view of the global energy crisis and its affordability due to very high operational cost. These all factors are compelling the employment of principles of ecological engineering for not only waste treatment but also for conserving biological communities in poor nations of the world.

The performance of various wetlands, land, and aquatic treatment were reviewed. Constructed wetlands are designed based on principles of natural wetlands, employing different aquatic plants like floating, submerged, and rooted macrophytes. The prominent feature of these treatment systems is extensive root system which helps to absorb various pollutants from their growth medium. The presence of various planktonic forms also aids in treatability of constructed wetlands. The constructed wetlands have been found useful in metals absorption, nutrients uptake (especially nitrogen and phosphorus), and reduction of BOD. Vermicomposting constructed wetlands have been found as useful in organic matter removal and it is a recent trend to include earthworms in wetlands for assisting the composting process as well as the quality of the end products. In addition, vermicomposting produces to be odor-free end products.

The unique adaptations of mangroves to stressed environments and a massive requirement for nutrients due to fast growth and high primary productivity, metabolism and yield which can be exploited for environmental remediation. The mangrove plants can modify their growth medium by excreting exogenous enzymes and can also affect microbial species composition and diversity by releasing oxygen into the rhizosphere that in turn indirectly influences enzyme activity and thus treatment process.

Land treatment systems comprise a possible alternative solution for wastewater management in cases where the constructions of conventional wastewater treatment plants are not afforded or other disposal option are not accessible. The terrestrial treatment systems present many variations in their treatability and composition. These systems rely on soil characteristics, the characteristics of wastewater, topography, the presence of additional media in soil, and so forth. This system comprises spray irrigation system, rapid infiltration, and overland flows. Soil aquifer treatment system uses soil as a filter, removing particles found in the applied water. Biological processes also take place during soil aquifer treatment by microorganisms in the soil, while chemical treatment is accomplished by natural processes as neutralization, reduction, and oxidation during which one substance or compound in the recycled water is removed, broken down, or transformed into another.

Natural aquatic systems work on the natural ecological principals where aquatic plants, algae, and other microbes absorb pollutants found in the wastewater to accomplish treatment. Aerobic, anaerobic, and facultative wastewater treatment ponds along their design characteristics were reviewed in current review. Stabilization pond is one of the frequent wastewater treatment methodologies where inexpensive land is available. Facultative ponds and land application processes can produce excellent quality effluent with smaller energy budgets.

## Figures and Tables

**Table 1:** Typical characteristics of plant species used in constructed wetlands (modified after Crites and Tchobanoglous [170], Reed [171]).

Characteristic	Bulrush	Cattail	Reeds
Distribution	Worldwide	Worldwide	Worldwide
Preferred temperature (°C)	16–27	10–30	12–23
Preferred pH range	4–9	4–10	2–8
Salinity tolerance (ppt*)	20	30	45
Root penetration (m)	*≈*0.6	*≈*0.3	*≈*0.4
Drought resistant	moderate	Possible	high
Growth	Moderate to rapid	Rapid	Very rapid

*ppt: parts per thousand.

**Table 2:** Comparison of some vermicomposting earthworm species in terms of the optimal and tolerable temperature ranges [119, 124, 172].

Species	Temperature ranges (°C)		Distribution
	Tolerated	Optimum	
*Eisenia * *fetida *	0–35	20–25	Temperate regions
*Eudrilus * *eugeniae *	9–30	20–28	Africa, India, North, and South America
*Perionyx * *excavatus *	9–30	15–30	Asia and Australia
*Eisenia * *veneta *	3–33	15–25	Europe

**Table 3:** Various plants species involved in wastewater treatment.

Plant species	Nature of waste	References
*Arundo donax* (reeds) *Eucalyptus botryoides *(Southern mahogany)	Primary effluents* Meat processing effluent	[173, 174]
*Eucalyptus camaldulensis *(red gum)*Eucalyptus ovata* (swamp gum) *Eucalyptus grandis* (rose gum)	Primary effluents*, storm water pond Meat processing effluent	[173–175]
*Eucalyptus globulus* (Tasmanian bluegum) *Eucalyptus cyanophylla *(blue leaved mallee)	Secondary effluent**, storm water pond	[175–177]
*Chloris gayana* (Rhodes grass)	Secondary effluent** enriched with nitrogen	[178]
*Eucalyptus robusta* (swamp mahogany)	Secondary effluent** enriched with nitrogen	[178]

*The liquid portion of wastewater leaving primary treatment like sedimentation but not biological oxidation.

**The liquid portion of wastewater leaving secondary treatment facility involving biological processes.

**Table 4:** Design features of terrestrial treatment system.

Feature	Slow rate	Rapid infiltration	Infiltration	Overland flow
Soil texture	Sandy loam	Sand and sandy	Sand to clayey, silty, loam, and clay loam	Clayey loam
Depth to	3 ft	3 ft	3 ft Not critical	Groundwater
Vegetation	Required	Optional	Not applicable	Required
Climatic restrictions	Growing season	None	None	Growing season
Slope	<20%, Not critical	Not applicable	2%–8% slopes cultivated land	<40%, uncultivated land

[179].

**Table 5:** Total annual energy for typical 1 mgd system including electrical plus fuel, expressed as 1000 kwh/yr [180].

Treatment system	Energy (1000 KwH/yr)
Rapid infiltration (facultative pond)	150
Slow rate, ridge, and furrow (facultative pond)	181
Overland flow (facultative pond)	226
Facultative pond + intermittent sand filter	241
Facultative pond + microscreens	281
Aerated pond + intermittent sand filter	506
Extended aeration + sludge drying	683
Extended aeration + intermittent sand filter	708
Trickling filter + anaerobic digestion	783
RBC + anaerobic digestion	794
Trickling filter + gravity filtration	805
Trickling filter + N removal + filter	838
Activated sludge + anaerobic digestion	889
Activated sludge + anaerobic digestion + filter	911
Activated sludge + nitrification + filter	1051
Activated sludge + sludge incineration	1440
Activated sludge + AWT	3809
Physical chemical advanced secondary	4464

These energy requirements were reported for meeting the four effluent quality standards, that is, BOD_5_, TSS, P, and N.
