# Corrigendum to “Natural Treatment Systems as Sustainable Ecotechnologies for the Developing Countries”

**DOI:** 10.1155/2018/4761769

**Published:** 2018-03-20

**Authors:** Qaisar Mahmood, Arshid Pervez, Bibi Saima Zeb, Habiba Zaffar, Hajra Yaqoob, Muhammad Waseem, Zahidullah, Sumera Afsheen

**Affiliations:** ^1^Department of Plant and Soil Sciences, University of Kentucky, Lexington, KY 40546-0091, USA; ^2^Department of Environmental Sciences, COMSATS Institute of Information Technology, Abbottabad 22060, Pakistan; ^3^Department of Biology, Allama Iqbal Open University, H-8, Islamabad 44000, Pakistan; ^4^Department of Zoology, University of Gujrat, Gujrat 50700, Pakistan

The article titled “Natural Treatment Systems as Sustainable Ecotechnologies for the Developing Countries” [1] was found to contain material from published work that was all cited. The authors apologize for not properly quoting and attributing the source of the wording. Details of the similarity are as follows.

- “Principles of Design and Operations of Wastewater Treatment Pond Systems for Plant Operators, Engineers, and Managers” [2]:
  - In the “Overview of Pond Systems for Wastewater Treatment” subsection, the paragraph “The primary costs associated with…energy requirements are minimal [164]” and most of the text beginning with “The advantages include reliable BOD5 removal…odors can be an intermittent problem [164].”
  - In the “Energy Requirements for Various Natural Treatment Systems” subsection, the paragraph “The available cost data for different types…can produce excellent quality effluent with smaller energy budgets.”
- “Seasonal and diurnal variations of temperature, pH and dissolved oxygen in advanced integrated wastewater pond system treating tannery effluent,” [3]:
  - In the “Design of Wastewater Pond Systems” subsection, the text beginning with “Wastewater ponds are natural systems whose biochemical…should always be considered [161].”
- “Implementation of earthworm-assisted constructed wetlands to treat wastewater and possibility of using alternative plants in constructed wetlands” [4]:
  - In the “Introduction” section, the paragraph “The conventional systems that may be…for those regions with limited resources [2].”
  - In the “Vermicomposting and Constructed Wetlands” subsection, great amount of the text beginning with “Vermicomposting principally employs earthworms…in animal and vegetable wastes” and some phrases from the text “They investigated the application of integrating… apply earthworms inside the wetlands [127].”
- “The use of free water surface constructed wetland to treat the eutrophicated waters of lake L'Albufera de Valencia (Spain)” [5]:
  - In the “Removal of Inorganics and Metals by CW” subsection, most of the text beginning with “Three free water surface constructed wetlands…but could be sent back in several years [92].”
- “Treatment of wastewater with slow rate systems: a review of treatment processes and plant functions” [6]:
  - In the “Land Treatment Systems” section, the text “Land treatment systems comprise a possible alternative solution…and maintenance costs [137]” and the paragraph “Recognizing the importance of wastewater…expected to increase.”
  - In the “Fundamental Processes” subsection, the paragraph “Slow rate systems purify the applied wastewater…toxic organics degradation/inactivation.”
  - In the “Vegetation of Terrestrial Systems” subsection, the paragraph “The primary criteria for vegetation selection…slow rate systems (SRS) worldwide.”
  - In the “Conclusion” section, the sentence “Land treatment systems comprise a possible alternative solution for wastewater management in cases where the constructions of conventional wastewater treatment plants are not afforded or other disposal option are not accessible.”
- “Iron and manganese in sediments of constructed wetlands with horizontal subsurface flow treating municipal sewage” [7]:
  - In the “Removal of Inorganics and Metals by CW” subsection, the paragraph “In 2008, concentrations of iron and manganese…sediment in the filtration bed [94].”
- “Potential use of mangroves as constructed wetland for municipal sewage treatment in Futian, Shenzhen, China” [8]:
  - In the “Role of Mangroves” subsection, the text “Mangroves are sole wetlands…for wastewater treatment [81, 88, 130]” and “A pilot-scale mangrove wetland…coliforms was also included [134].”

## References

[1] Mahmood Q., Pervez A., Zeb B. S. (2013). Natural treatment systems as sustainable ecotechnologies for the developing countries. *BioMed Research International*.

[2] EPA. (2011). *Principles of Design and Operations of Wastewater Treatment Pond Systems For Plant Operators, Engineers and Managers*.

[3] Tadesse I., Green F. B., Puhakka J. A. (2004). Seasonal and diurnal variations of temperature, pH and dissolved oxygen in advanced integrated wastewater pond system® treating tannery effluent. *Water Research*.

[4] Chiarawatchai N. (2010). *Implementation of earthworm-assisted constructed wetlands to treat wastewater and possibility of using alternative plants in constructed wetlands*.

[5] Martín M., Oliver N., Hernández-Crespo C., Gargallo S., Regidor M. C. (2013). The use of free water surface constructed wetland to treat the eutrophicated waters of lake L'Albufera de Valencia (Spain). *Ecological Engineering*.

[6] Paranychianakis N. V., Angelakis A. N., Leverenz H., Tchobanoglous G. (2006). Treatment of wastewater with slow rate systems: A review of treatment processes and plant functions. *Critical Reviews in Environmental Science and Technology*.

[7] Vymazal J., Švehla J. (2013). Iron and manganese in sediments of constructed wetlands with horizontal subsurface flow treating municipal sewage. *Ecological Engineering*.

[8] Yang Q., Tam N. F. Y., Wong Y. S. (2008). Potential use of mangroves as constructed wetland for municipal sewage treatment in Futian, Shenzhen, China. *Marine Pollution Bulletin*.

